# The Role of Chronic Inflammation in Polycystic Ovarian Syndrome—A Systematic Review and Meta-Analysis

**DOI:** 10.3390/ijms22052734

**Published:** 2021-03-08

**Authors:** Shaimaa Aboeldalyl, Cathryn James, Emaduldin Seyam, Emad Moussa Ibrahim, Hossam El-Din Shawki, Saad Amer

**Affiliations:** 1 Academic Unit of Translational Medical Sciences, School of Medicine, Royal Derby Hospital Centre, University of Nottingham, Derby DE22 3DT, UK; shaimaa.aboeldalyl@nhs.net; 2 Faculty of Medicine, Obstetrics and Gynaecology, University of Minia, Minia 61519, Egypt; eemsseyam@yahoo.com (E.S.); ibrahimmemo@hotmil.com (E.M.I.); hossamshawki2019@yahoo.com (H.E.-D.S.); 3 University Hospitals of Derby and Burton NHS Foundation Trust, Library & Knowledge Service, Derby DE22 3NT, UK; cathrynjames1961@gmail.com

**Keywords:** polycystic ovarian syndrome, chronic inflammation, inflammatory markers, C-reactive protein, insulin resistance, obesity

## Abstract

Although the current literature associates polycystic ovarian syndrome (PCOS) with chronic inflammation, the evidence for this link remains inconclusive and its causal nature remains unclear. The purpose of this systematic review was to assess the inflammatory status in PCOS women and to determine whether it is related to PCOS or to its associated adiposity. We searched electronic databases including PUBMED, EMBASE and MEDLINE, SCOPUS, DynaMed plus, TRIP, ScienceDirect and Cochrane Library, for studies investigating C-reactive protein (CRP) and other inflammatory makers in PCOS women versus healthy controls. Quality and risk of bias for selected studies were assessed using the modified Newcastle–Ottawa scale. CRP data were extracted and pooled using RevMan for calculation of the standardized mean difference (SMD) and 95% confidence interval (CI). Eighty-five eligible studies were included in the systematic review, of which 63 were included in the meta-analysis. Pooled analysis of the 63 studies revealed significantly higher circulating CRP in PCOS women (*n* = 4086) versus controls (*n* = 3120) (SMD 1.26, 95%CI, 0.99, 1.53). Sensitivity meta-analysis of 35 high quality studies including non-obese women showed significantly higher circulating CRP in PCOS women versus controls (SMD 1.80, 95%CI, 1.36, 2.25). In conclusion, circulating CRP is moderately elevated in PCOS women independent of obesity, which is indicative of low-grade chronic inflammation.

## 1. Introduction

Polycystic ovary syndrome (PCOS) is the most common endocrine disorder affecting women with a prevalence of 15–20% amongst reproductive age women according to the Rotterdam diagnostic criteria [1,2,3], 83% of women with anovulatory infertility [4] and 89% of hyperandrogenic women [5]. The syndrome is characterized by a varied combination of clinical (anovulation and hyperandrogenism), biochemical (excess serum luteinizing hormone and androgen concentrations) and ovarian morphological (polycystic ovaries) features [6]. Hyperandrogenism has been reported in about 80% of PCOS women [1] with hirsutism being the most common androgenic feature affecting 70% of cases [7]. Although insulin resistance is not one of the Rotterdam diagnostic criteria, it has been reported in up to 70% of women with PCOS [8,9,10]. The syndrome is well-known to negatively affect the psychological and emotional wellbeing of women due to its distressing reproductive and androgenic symptoms [11].

PCOS has been associated with significant long-term metabolic and cardiovascular morbidities, possibly due to insulin resistance [6]. It has also been associated with chronic low-grade inflammation, which is thought to contribute to the long-term cardiovascular risks [12,13].

Despite its high prevalence, the underlying mechanisms of PCOS remain uncertain. A significant body of evidence implicates androgen excess and insulin resistance in its pathogenesis. Several studies have reported a positive correlation between chronic low-grade inflammation and hyperandrogenism in PCOS women. However, the causal nature of this association remains uncertain [12].

Our group and others have recently shown increased androgen synthesis in subcutaneous adipose tissue (SAT) that could contribute to PCOS related hyperandrogenaemia [14,15]. Adipose tissue is also recognised as an important site for excess production of pro-inflammatory mediators with subsequent chronic inflammation in women with PCOS as well as other insulin resistant conditions such as obesity [16]. Activation of inflammatory pathways in adipocytes has been found to impair triglyceride storage with increased release of free fatty acids, which could induce insulin resistance [17]. The nature of the association between hyperandrogenaemia, insulin resistance and chronic inflammation remains to fully understood.

Low grade chronic inflammation is often assessed primarily by measuring serum levels of C-reactive protein (CRP) in addition to several other inflammatory markers. CRP is a liver-derived acute phase protein produced in response to release of interleukin-6 (IL-6) from activated immune cells such as macrophages and from adipocytes [18,19]. Increased serum CRP level has been strongly linked to long-term health risks in women with obesity and type 2 diabetes and cardiovascular disease (CVD) [20,21,22].

The status of circulating CRP and other inflammatory markers in PCOS women has previously been investigated in many studies with conflicting results. While some studies reported elevated CRP levels in PCOS women [23,24], others showed no differences in circulating CRP between women with and without PCOS [25,26]. A systematic review and meta-analysis involving 31 studies was published on this topic in 2011 providing evidence for increased circulating CRP (96% higher than controls) and other inflammatory markers in PCOS [27]. However, since that review there have been over 100 published studies assessing CRP in PCOS women utilising more advanced CRP assays. The purpose of the current meta-analysis was therefore to assess the inflammatory status as determined by circulating CRP in women with PCOS based on the most recent studies.

## 2. Results

### 2.1. Search Results

**Figure 1:**
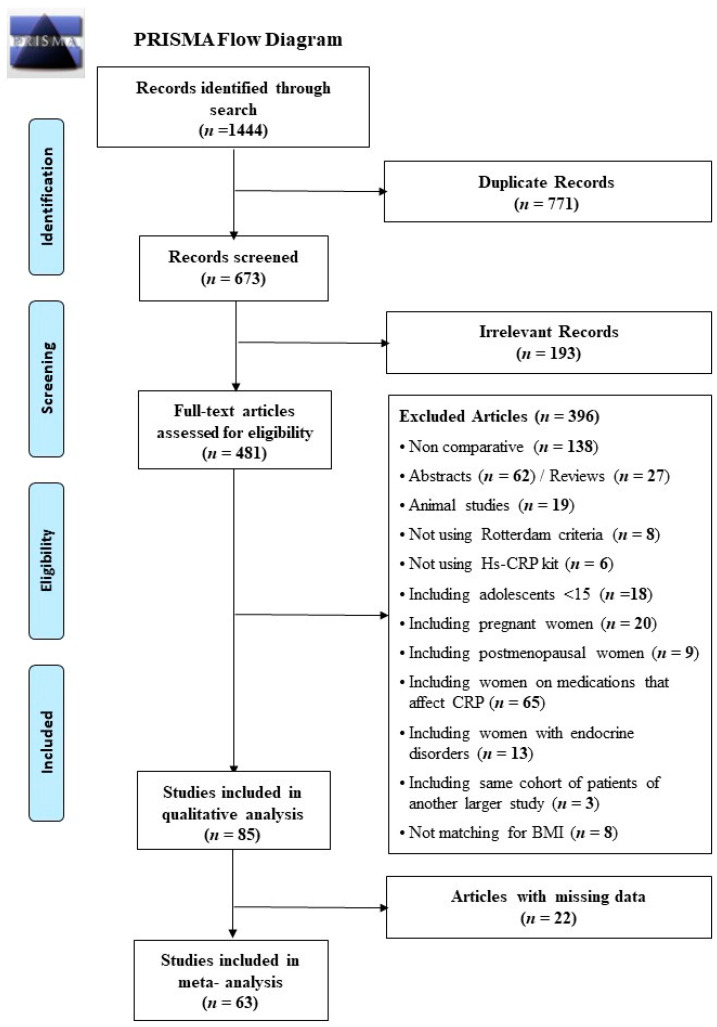
PRISMA Flow chart.

Our search yielded 1444 articles, which came down to 673 after exclusion of duplicates. Screening of these 673 articles revealed 193 irrelevant articles, which were excluded. The full texts of the remaining 481 papers were carefully reviewed for eligibility criteria. Of these, 385 articles did not meet the inclusion criteria and were excluded. Three further articles [28,29,30] were excluded as each of them involved participants who were included in another larger article by the same research group [31,32,33]. The authors of these studies were contacted to ascertain if their cohorts of participants were different in each study, but no reply was received. Eight other articles neither matched their PCOS women and healthy controls for BMI nor adjusted their analysis for BMI differences [34,35,36,37,38,39,40,41]. In total, 396 articles were excluded and 85 met the inclusion criteria and were included in this review (Figure 1).

#### 2.1.1. Risk of Bias and Quality Assessment of Selected Studies

Table 1 shows the results of quality scores of all 85 studies in this review.

#### 2.1.2. Excluded Studies

Of the 396 excluded articles, 138 were not comparative studies, 62 were abstracts with incomplete data, 27 were reviews, 19 were animal studies, and 150 had other exclusion criteria as detailed in Figure 1.

#### 2.1.3. Included Studies

Characteristics of the 85 included studies are shown in Table 1. Of these, 45 studies provided data on other inflammatory markers such as IL-6, TNF-α, adiponectin, etc., as summarised in Table 2.

Mean ± SD CRP values were not provided in 22 studies [42,46,60,62,63,65,66,68,72,73,78,82,83,92,93,99,108,110,112,118,119,121]. Authors of these articles were contacted to provide the missing data, but no response was received despite several reminders. These studies were included in the systematic review but excluded from the meta-analysis. The remaining 63 studies had all the required CRP data and were used for meta-analysis of CRP results (Figure 1).

#### 2.1.4. Study Design

Seventy-eight of the included articles were case–control cross-sectional studies. The remaining seven studies were interventional studies investigating the effect of different therapeutic interventions on inflammatory markers in PCOS women versus controls [43,48,50,85,90,109,122]. All these studies provided baseline data for PCOS women versus controls, which were suitable for this systematic review.

#### 2.1.5. Participants

All studies used appropriate selection for their participants, which met our inclusion criteria. Rotterdam ESHRE/ASM criteria were used in all 85 studies for the diagnosis of PCOS. All participants were in childbearing age between 15–45 years, had no endocrinological disease, were not pregnant at time of participation and were not on any medication that might affect the level of inflammatory markers in the preceding three months.

#### 2.1.6. CRP Kits

Several high sensitivity (hs) CRP assays have been utilised in different studies as illustrated in Table 1. The most used assay was immunoturbidimetric assay (ITA) (44 studies), followed by the immunonephelometric assay (INA) (13 studies). Other CRP assays include hs-ELISA (nine studies), chemiluminescence immunoassay (ECLIA) (six studies), hs-EIA (one studies), latex immunoassay (LIA) (one study), and mass spectrometry (two studies). The assay was not specified in nine studies, but the authors stated that they used a high sensitivity CRP assay (Table 2).

Hs-ELISA is known for its relative ease of use, accuracy, and low cost. Its main drawback is its inflexibility to reagents and limited accuracy in markedly low concentrations (>1 μg/L). Only two studies used mass spectrometry, which is the most accurate measurement. ITA and INA, which are the most used assays, provide excellent sensitivity with a detection time of < 1h [124]. However, these methods require relatively expensive, complex, and require an inflexible set of reagents.

### 2.2. Systematic Review

#### 2.2.1. CRP Results

Serum CRP level was measured in 85 comparative studies (*n* = 9880), of which 53 reported significantly higher levels in PCOS women (*n* = 5656) compared to healthy controls (*n* = 4224). Details of CRP results of all 85 studies are summarised in Table 1. BMI of participants was either matched on selection (79 studies) or adjusted for during statistical analysis (six studies) concluding that the difference in CRP was attributed to PCOS status rather than obesity (Table 1). Age was matched for all studies apart from two studies [32,68].

Five other studies reported a statistically significant increase in serum CRP level among the PCOS women (*n* = 327) compared with healthy controls (*n* = 276), but after adjustment for BMI the difference was no longer statistically significant [65,67,75,118,119].

Two other studies showed a higher trend of serum CRP levels in PCOS women (*n* = 161) compared to healthy controls (*n* = 123), but this did not reach statistical significance [52,76]. The remaining 25 studies failed to demonstrate any statistically significant difference in serum CRP levels between PCOS women and healthy controls. Of these, seven studies reported statistically significant increase in other inflammatory markers as described below [49,50,59,67,69,85,86].

To further investigate the effect of obesity as a main confounder on inflammatory status, we separately looked at eleven studies that stratified their participants according to BMI [51,55,59,66,78,87,96,98,100,115,122] and two studies that included only obese participants only [45,110] (Table 1). Ten of the eleven studies stratifying PCOS group according to BMI, revealed significantly higher circulating CRP in obese/overweight versus non-obese PCOS women, while one study showed no difference [96]. On the other hand, eight studies comparing between BMI strata in the control group reported higher CRP levels in the higher BMI group [51,59,78,87,98,100,115,122], while two studies revealed no difference [55,96]. Comparison between different BMI strata between PCOS women and controls is provided in the meta-analysis section below.

#### 2.2.2. IL-6 Results

Circulating IL-6 was measured in 16 articles, of which five reported significantly higher levels in PCOS women compared to healthy controls (Table 3) [53,55,70,73,116]. The remaining 11 studies found no significant difference in circulating IL-6 between PCOS and non-PCOS women [45,48,49,50,56,59,63,67,82,92,96].

#### 2.2.3. TNF-α Results

Serum TNF-α concentration was measured in 12 studies with conflicting results (Table 3). While six studies reported higher serum TNF-α levels in PCOS women versus healthy controls [50,55,63,70,103,116], six studies showed no difference between the two groups (Table 3) [45,56,67,83,92,96].

#### 2.2.4. White Cell Count (WCC)

Eight studies (*n* = 1026) assessed WCC in PCOS women (*n* = 610) versus healthy controls (*n* = 416) [46,47,70,77,85,87,98,101]. Of these, seven studies reported significantly higher WCC in PCOS versus control participants, while one study showed no difference between the two groups (Table 3) [70].

#### 2.2.5. Adiponectin

Eight studies including 892 participants assessed circulating adiponectin, with six showing significantly lower levels in PCOS women (*n* = 383) versus controls (*n* = 355) [50,51,63,73,112,116,123] and two reporting no difference between the two groups (PCOS, *n* = 100; control, *n* = 54) (Table 3) [45,83].

### 2.3. Meta-Analysis

#### 2.3.1. Primary Outcome: CRP

##### Overall CRP Pooled Analysis

**Figure 2:**
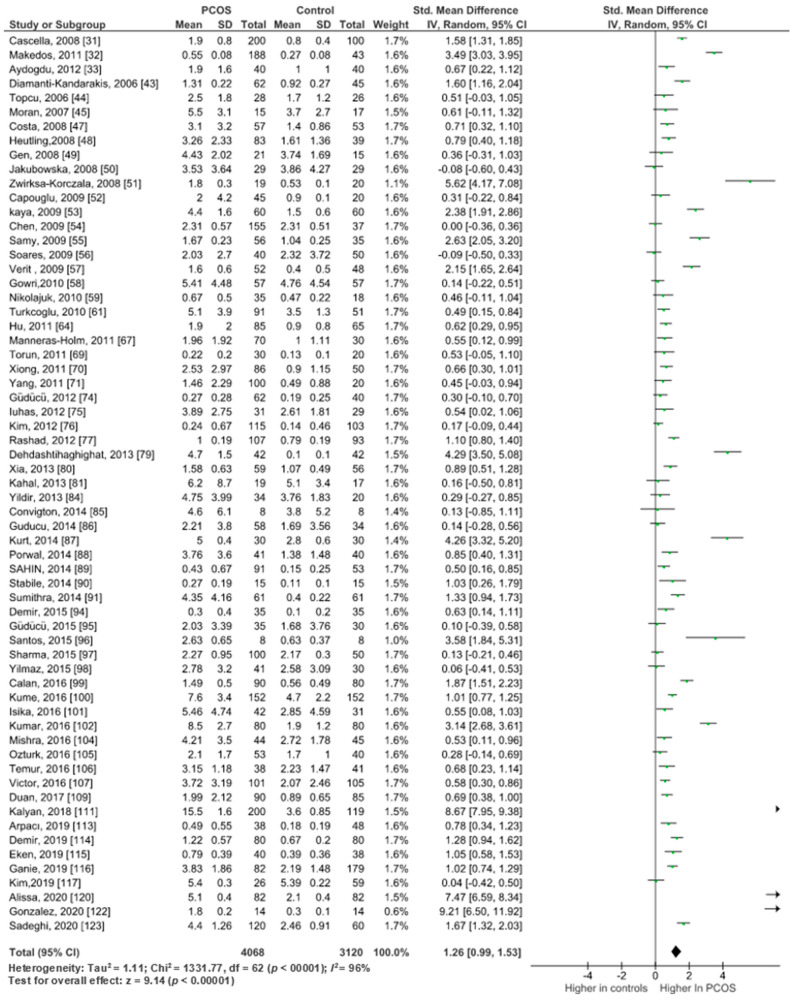
Overall CRP pooled analysis of 63 studies.

Pooled analysis of 63 studies (*n* = 7206) with relevant data, showed significantly higher serum CRP levels in PCOS women (*n* = 4086) versus controls (*n* = 3120) (SMD 1.26, 95% CI, 0.99, 1.53; z = 9.60; *p* = 0.00001; *I*^2^ = 96%). Heterogeneity between studies was high (Figure 2).

##### Studies Using mg/L as a Unit of CRP Measurement

Data from 58 studies (*n* = 6842) using mg/L for CRP measurement showed significantly higher circulating CRP in PCOS women (*n* = 3884) versus controls (*n* = 2958) (SMD 1.32, 95% CI, 1.03, 1.61; z = 8.89; *p* = 0.00001; *I*^2^ = 96%).

##### Sensitivity Analysis of High-Quality Studies Including Only Non-Obese Participants (BMI < 30)

**Figure 3:**
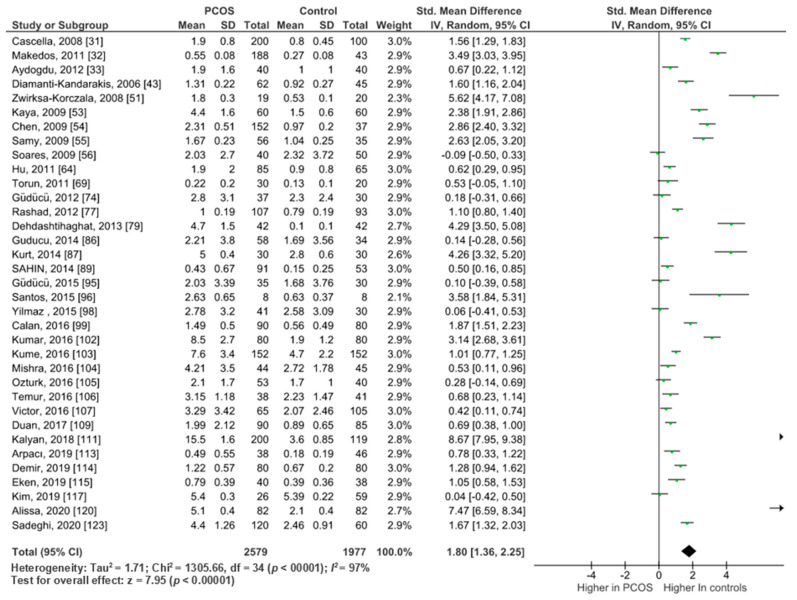
CRP sensitivity analysis of 35 high quality studies including non-obese women.

This analysis included 35 studies scoring ≥7 on the modified New-castle Ottawa scale and providing data for women with BMI < 30 kg/m^2^ (*n* = 4556). We only analysed data of non-obese participants to remove the known confounding effect of obesity on CRP. Pooled analysis from these studies still showed significantly higher circulating CRP in PCOS women (*n* = 2579) compared to controls (*n* = 1977) (SMD 1.80, 95%CI, 1.36, 2.25; z = 7.97; *p* < 0.00001; *I*^2^ *=* 97%). (Figure 3).

##### Studies Including Only Obese Participants (BMI > 30)

**Figure 4:**
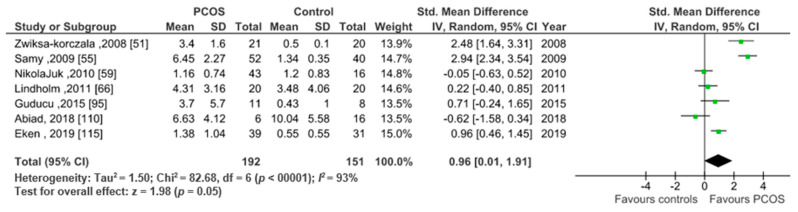
CRP subgroup analysis of 7 studies including obese women.

This analysis included seven studies presenting CRP data (mean ± SD) for obese participants (*n* = 343). Pooled analysis revealed significantly higher CRP levels in 192 PCOS women versus 151 healthy controls (SMD 0.96, 95% CI, 0.01, 1.91; z = 1.98; *p* = 0.05; *I*^2^ = 98%). (Figure 4). The magnitude of this difference was smaller than that between of normal weight groups.

#### 2.3.2. IL-6 Meta-Analysis.

**Figure 5:**
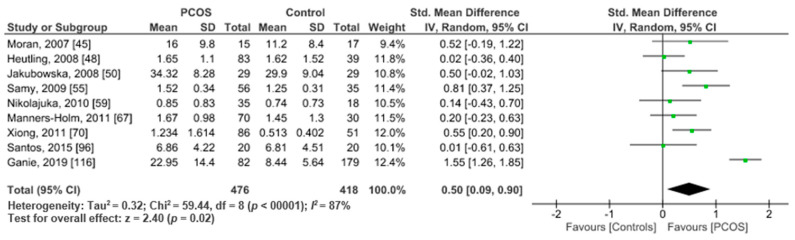
IL-6 metanalysis of 9 studies.

Nine studies involving 894 participants presented IL-6 data suitable for meta-analysis. Pooled analysis of these studies showed significantly higher serum IL-6 levels in 476 PCOS women versus 418 controls (SMD 0.50, CI 95%, 0.09, 0.90; z = 2.40; *p* = 0.02; *I*^2^ = 87%) (Figure 5).

#### 2.3.3. TNF-α Meta-Analysis

**Figure 6:**
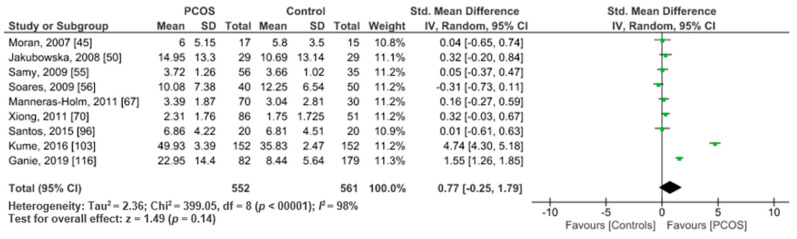
TNF-α metanalysis of 9 studies.

TNF-α data were available from nine studies comparing PCOS women (*n* = 552) versus healthy controls (*n* = 561). Pooled analysis revealed no significant difference between the two groups (SMD 0.77, 95% CI, −0.25, 1.79; z = 1.49; *p* = 0.14; *I*^2^ = 98%) (Figure 6).

#### 2.3.4. Adiponectin Meta-Analysis

**Figure 7:**
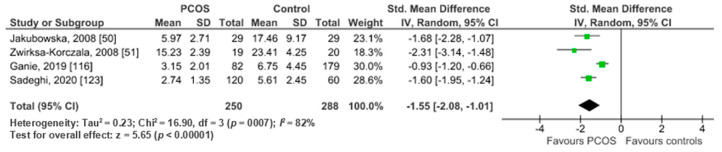
Adiponectin metanalysis of 4 studies.

Pooled analysis of four studies (*n* = 538) revealed significantly lower serum adiponectin levels in PCOS women (*n* = 250) compared to healthy controls (*n* = 288) (SMD −1.48 95% CI; −2.48, −0.14; z = 2.91; *I*^2^ = 87%) (Figure 7)

## 3. Discussion

To the best of our knowledge, this is the largest systematic review and meta-analysis with nearly 10,000 participants, that investigates the chronic inflammatory status in PCOS women. We have analysed 85 studies (*n* = 9880) comparing circulating CRP and other inflammatory markers in PCOS women (*n* = 5656) versus healthy controls (*n* = 4224). Although, there was some conflicting results with CRP from different studies, most publications reported significant elevation of circulating CRP in PCOS women compared to healthy controls (SMD 1.37). Furthermore, sensitivity meta-analysis of CRP data in non-obese women from 35 studies with low risk of bias (*n* = 4556) still revealed a significantly elevated CRP in PCOS women versus healthy controls with an SMD of 1.80. On the other hand, subanalysis of CRP data in obese women revealed a much less pronounced CRP elevation (SMD 0.96) in PCOS women. These findings support the hypothesis that that the chronic inflammation observed in PCOS women is independent of obesity.

While meta-analysis of IL-6 revealed significantly higher levels in PCOS women, pooled analysis of TNF-α showed no significant difference between the two groups. On the other hand, most studies reported that WCC was consistently elevated and adiponectin was consistently reduced in PCOS women versus controls providing more evidence for an ongoing inflammatory process in this population.

### 3.1. Comparison with Previous Studies

Our results are consistent with the previous systematic review published in 2011 by Escobar-Morreale et al., which reported a two-fold increase in CRP [27]. However, unlike this previous review, which included studies adopting different sets of diagnostic criteria for PCOS, we adhered only to the universally accepted Rotterdam criteria thereby reducing heterogeneity. Furthermore, Escobar-Morreale et al. only used PubMed search engine to identify studies, while we have applied a more comprehensive search including all available electronic databases as detailed above. In addition, we performed risk of bias assessment of selected articles to identify high quality studies for a sensitivity analysis, which is lacking in the previous review. Moreover, our much larger number of PCOS participants allowed us to undertake sub-analysis for non-obese PCOS women, thereby eliminating the confounding effect of obesity on the inflammatory status of participants. Additionally, our review included more recent studies, which utilised advanced CRP assays with much improved sensitivity and accuracy. We also assessed the status of the anti-inflammatory factor adiponectin in PCOS women, which was not evaluated in the previous review.

Although, 32 of the 85 studies included in our review did not show a significant elevation in circulating CRP, some of these showed a trend of higher CRP, which did not reach statistical significance, possibly due to the small numbers included (type II error). Furthermore, several studies showing no increase in CRP reported elevation of other markers such as IL-6, TNF-α other ILs and WCC.

A possible explanation for the discrepancies in CRP results between studies could be the variation in phenotypes of PCOS women included in different studies. For instance, Daskalopoulos et al. reported that PCOS women with no hyperandrogenism had CRP levels comparable to healthy controls [93]. On the other hand, PCOS women with full features of the syndrome had a 3-fold increase in levels of CRP compared to PCOS women with normal androgen levels [93]. This is further supported by the hypothesis that excess androgens have been implicated as a possible underlying cause for chronic inflammation as discussed below [13].

### 3.2. Inflammatory Status in PCOS Women

Despite some discrepancy in the literature as discussed above, our meta-analysis provides strong evidence for a chronic inflammation in women with PCOS as evidenced by the significant elevation of circulating CRP in most reviewed studies. Notably, the elevation in circulating CRP is only modest with levels not exceeding 5.0 mg/L in PCOS women in most studies indicating low-grade inflammation. The status of other main inflammatory markers including IL-6 and TNF-α is still not clear with limited and conflicting data. However, the increased CRP provides an indirect evidence of increased IL-6 and TNF-α, which are the main mediators of CRP production in the liver.

Our findings also confirm that the obesity-related increase in CRP is further enhanced by the presence of PCOS. However, whilst the elevation in circulating CRP in PCOS is evident in normal weight women, it is less pronounced in obese PCOS when compared to obese controls. This is consistent with several previous reports [42,125]. This led Gonzalez to hypothesize that CRP elevations attributable to PCOS may be obscured in the presence of obesity [126]. This suggests that CRP level may be a less sensitive single indicator of chronic inflammation in obese PCOS women. [126].

In addition to the increased inflammatory markers, our review has found reduced adiponectin, which is a well-known anti-inflammatory factor, in PCOS women. Some studies have also reported a decrease in Pentraxin -3 (PTX3) [30,86], which is structurally related to CRP and plays a regulatory role in inflammation with a possible protective effect against atherosclerosis and cardiovascular risks [127]. All these findings indicate that the ongoing inflammation in PCOS is a double-sided process with increased proinflammatory factors as well as reduced inflammatory protective factors.

More evidence of the inflammatory status in PCOS independent of obesity comes from two studies reporting the effect of weight loss on inflammatory markers in obese women [45,110]. Whilst weight loss was associated with beneficial lowering effect on inflammatory markers in both PCOS and non-PCOS obese women, this effect was less evident and slower in the PCOS group. These findings provide further evidence supporting the hypothesis that chronic inflammation is related to PCOS pathology independent of obesity.

### 3.3. Mechanisms of Inflammation in PCOS

The exact mechanism of inflammation in PCOS women is largely unknown. Excess adiposity, which is very common in PCOS women is a well-known proinflammatory condition, in which hypertrophied adipocytes and adipose tissue-resident immune cells contribute to chronic low-grade inflammation [128]. However, other factors must account for inflammation in lean PCOS women and for the greater than expected inflammation in obese PCOS women.

There is limited evidence suggesting a genetic basis for the chronic low-grade inflammation observed in PCOS women. Few studies have reported an association between PCOS and proinflammatory genotypes including those encoding TNF-α, type 2 TNF receptor and IL-6 [129,130,131].

PCOS-associated hyperandrogenism has also been suggested as a possible underlying cause for adipose tissue inflammation. Deligeoroglou et al. [13] hypothesized that androgen-induced adipocyte hypertrophy causes compression of stromal vessels leading to tissue hypoxia, which is known to trigger inflammation.

### 3.4. The Role of CRP in PCOS Pathogenesis

A significant body of evidence implicates androgen excess and insulin resistance in the pathogenesis of PCOS. Both androgen excess and insulin resistance are strongly associated with chronic inflammation. Based on this and on the findings of our meta-analysis, we can conclude that androgen excess, insulin resistance and chronic inflammation, which are all interlinked are strongly associated with PCOS. The exact interaction between these three pathological entities and their exact role in the pathogenesis PCOS remain to be further elucidated.

#### 3.4.1. Chronic Inflammation and Hyperandrogenism

Current evidence strongly suggests a key role of adipose tissue in mediating chronic inflammation in obesity and diabetes [132,133]. In obesity, hypertrophied adipocytes and adipose tissue-resident immune cells (lymphocytes and macrophages) are the main sources of increased circulating proinflammatory cytokines. Furthermore, obesity-associated chronic inflammation, termed “metabolic inflammation,” is considered crucial in the pathogenesis of insulin resistance and type 2 diabetes (T2D) [128,133,134,135,136]. Given the well-known link between obesity, T2D and PCOS, it is possible to hypothesis that adipose tissue inflammation may play a crucial role in PCOS pathogenesis.

We and others have previously provided evidence for adipose tissue as a possible source of excess androgen production in women with PCOS [14,15,137]. Furthermore, several studies have reported a positive correlation between chronic low-grade inflammation and hyperandrogenism in PCOS [93]. Given the fact that adipose tissue is the site for both chronic inflammation and excess androgen production in PCOS, the causal relation between these two processes remains largely uncertain with limited conflicting literature data [12,13]. While Deligeoroglou et al. [13] reported that androgen excess could trigger inflammation (as explained above), González et al. [126] found that diet-induced inflammation in PCOS women has been shown to directly invoke hyperandrogenism. The interaction between hyperandrogenaemia and adipose tissue inflammation in PCOS therefore remains to be further elucidated. Most previous research has focused on circulatory markers of inflammation in PCOS women with no studies involving adipose tissue [126]. Given the well-known role of adipose tissue in chronic inflammation and excess androgen production, it will be crucial to study the inflammatory status in this tissue in PCOS in future research [132].

#### 3.4.2. Chronic Inflammation and Insulin Resistance

Insulin resistance has been reported in up to 70% of PCOS women [138], and insulin sensitivity is reduced by ~40% in PCOS women, independent of obesity [139]; although obesity further impairs insulin metabolism. The role of adipose tissue chronic inflammation in the development of insulin resistance is well established in obesity and T2D [133]. Two main adipose tissue-derived inflammatory cytokines have been implicated in insulin resistance including TNF-*α* and IL-6. TNF-*α*, which is primarily secreted by adipose tissue-resident macrophages, was the first inflammatory cytokine to be implicated in the induction of insulin resistance [8,15,16]. TNF-*α* levels are positively correlated with other markers of insulin resistance [19]. In rodents, obese mice lacking either TNF-α or its receptor show protection against the development of insulin resistance [17]. The main mechanism by which TNF-α induces insulin resistance is by triggering post-receptor serine phosphorylation of insulin receptor substrate-1 (IRS-1) thereby interfering with the insulin signalling pathway [22]. In addition, TNF-α is also known to promote lipolysis and the secretion of free fatty acids, which contribute to an increase in hepatic glucose production [23]. It also downregulates adiponectin [26], which is an adipocyte-derived hormone that contributes to the maintenance of peripheral glucose and lipid homeostasis [27].

On the other hand, the role of IL-6 in insulin resistance is less clear with conflicting literature data. It has been hypothesized that the effect of IL-6 on insulin metabolism varies according to the duration of IL-6 elevation (transient or chronic), the type of tissue and the metabolic state [133]. For instance, during exercise, a transient elevation of IL-6 increases glucose uptake in the skeletal muscle as well as having an anti-inflammatory effect [140]. On the other hand, in adipose tissue and liver, IL-6 exerts proinflammatory activities and induces insulin resistance by upregulating suppressor of cytokine signalling 3 (SOCS3), which is a protein that binds to and inhibits insulin receptor [141]. Additionally, it negatively regulates IRS-1 phosphorylation and transcription [142,143].

Based on the above, although the exact causal relation between chronic inflammation, insulin resistance and hyperandrogenism in PCOS is still unclear, there seems to be a vicious circle triad between these three elements of PCOS [112]. One possible hypothesis is that PCOS-related hyperandrogenism leads to adipose tissue inflammation with subsequent dysregulated adipokine secretion resulting in insulin resistance [112].

### 3.5. Limitations and Strengths

The main limitation in this review is the high heterogeneity between studies and the small size of many of the reviewed studies. Heterogeneity is mainly due to the variation in laboratory methods used to measure CRP and other markers, the variation in age and BMI of participants and the possible variation in PCOS phenotypes. To minimise heterogeneity, we carried out a sensitivity analysis of high-quality studies including only non-obese participants.

The main strength of the review is the large number of participants approaching 10,000. In all studies, CRP was measured early morning after an overnight fast. This helps to avoid the effect of circadian rhythm and the effect of food on CRP levels.

### 3.6. CRP and Cardiovascular Risk

According to the American Heart Association, elevated serum hs-CRP level is well-established as a prognostic marker of cardiovascular disease with a cut-off value of 3 mg/L that can detect patients at high risk, while a level of 1–3 mg/L is associated with a moderate risk [144,145]. Although most studies in our review reported elevated circulating CRP, only 35 of the 85 reviewed studies reported levels mean or median levels higher than 3 mg/L. Notably, the wide variation in CRP levels in different studies may limit our interpretation of the results. Nevertheless, measuring CRP may be considered in addition to other cardiovascular prognostic markers in PCOS women to identify those with CRP levels >3 mg/L. Furthermore, it may be necessary to monitor PCOS women with increased CRP (>3 mg/L) to detect an upward trend of CRP that would identify high-risk patients [144]. However, the marked biological variation should be taken into consideration when interpreting repeated CRP measurement.

### 3.7. Implications for Treatment and Future Research

It is tempting to hypothesize that drugs targeting chronic inflammation may reduce PCOS-associated cardiovascular risks. Preliminary data suggest that insulin sensitizing agents (e.g., metformin) may improve the inflammatory status in PCOS [43,90,146]; but the evidence is still inconclusive. Further research is required to understand the exact mechanisms and role of chronic inflammation in PCOS.

Oral contraceptive pills (OCPs), which are commonly used in PCOS, have recently been found to increase serum CRP levels according to a recent systematic review [147]. We may therefore need to practice some caution when prescribing OCPs to PCOS women or to consider alternative treatment options. Further research is required to assess this risk further.

## 4. Materials and Methods

This study has been conducted in line with the Preferred Reporting Items for Systematic Reviews and Meta-Analysis (PRISMA) guidelines and was prospectively registered with PROSPERO (registration number CRD42018085211).

### 4.1. Eligibility Criteria for Study Selection

We considered all studies comparing serum CRP concentrations between PCOS women diagnosed according to ESHRE/ASRM Rotterdam criteria [6] versus healthy controls. Only studies using high sensitivity CRP (hs-CRP) assays and matching and/or adjusting for BMI and age were included. In addition, the review included studies conducted on humans, written in English language, and involving non-pregnant women aged 15–45 years who were drug naïve and with no history of any disorder that might affect their inflammatory markers.

If more than one article by the same research group were identified, only the study with the larger sample size was included in the meta-analysis to avoid duplication of cases.

### 4.2. Outcome Measures

#### 4.2.1. Main Outcome

Serum CRP concentrations in PCOS women compared to controls.

#### 4.2.2. Secondary Outcome Measures

These included other proinflammatory markers (e.g., IL-6, TNF-α, IL-18) and the anti-inflammatory factor adiponectin measured in the serum of PCOS women and compared to controls.

### 4.3. Search Strategy

PUBMED, EMBASE and MEDLINE, SCOPUS, Dynamed, TRIP, scienceDirect and Cochrane Library were systematically searched starting from January 2000 to March 2020 for pertinent studies; A Combination of these search terms was used ((exp “POLYCYSTIC OVARY SYNDROME”/OR exp “POLYCYSTIC OVARY SYNDROME”/di OR (Polycystic Ovary Syndrome).ti,ab OR ((Polycystic AND Ovary) AND Syndrome).ti,ab OR (Polycystic Ovar*).ti,ab OR (Polycystic Ovar* Disease).ti,ab OR (PCOS).ti,ab) AND (((exp INFLAMMATION/OR (Inflammation).ti,ab) AND (exp BIOMARKERS/OR (Biomarkers).ti,ab)) OR (exp “C-REACTIVE PROTEIN”/OR (C Reactive Protein).ti,ab))) [DT FROM 2000] [Languages English]1.

The search was conducted by a clinical Librarian (LS) and repeated by a second independent Librarian. Further manual search of references of selected articles was conducted to identify eligible studies.

### 4.4. Screening and Selection of Retrieved Studies

Titles and abstracts of the articles retrieved by the above search were screened for relevance. Full texts of the relevant articles were further evaluated carefully, and eligible studies were selected according to the inclusion and exclusion criteria. This process was conducted by two independent reviewers (SA and SA) and in cases of disagreement, a consensus was reached after discussion between the two reviewers.

### 4.5. Assessment of Quality and Risk of Bias

Modified New-Castle Ottawa scale was used for assessment of non-randomized trials as recommended [148,149]. Three main aspects were evaluated in each study namely selection, comparability, and outcome with maximum score of four, two and three, respectively. Selection was assessed on basis of accurate definition of cases, inclusion of series of cases, inclusion of community control and power calculation. Studies were awarded a maximum of two stars in comparability if proper matching and/or adjustment to main confounders—namely age and BMI of participants-was ensured. Three aspects of outcome were evaluated which were timing of blood sampling following eight to twelve hours of fasting, use of validated laboratory methods to detect inflammatory markers and clear statistical comparisons between PCOS and control groups.

We included all retrieved eligible studies in our initial overall analysis. This was then followed by a sensitive analysis including only high-quality studies scoring at least 7 on the New-Castle Ottawa scale.

### 4.6. Data Extraction and Analysis

Data (mean ± sd) were extracted from the individual articles including age, BMI, CRP, and TNF-α, IL-6, and adiponectin. These data were entered into the RevMan software (Review Manager, version 5.1, The Cochrane Collaboration, 2011; The Nordic Cochrane Centre, Copenhagen, Denmark) for meta-analysis. The standard mean difference (SMD) in CRP, TNF-α, IL-6 and adiponectin between PCOS women and healthy controls was calculated. Statistical heterogeneity between studies was assessed by I-squared (*I*^2^) statistics and Chi square test. *I*^2^ values of ≥50% or chi square analysis larger than its degree of freedom was indicative of high heterogeneity [150]. When heterogeneity was significant, a random-effect model was used for meta-analysis. Fixed effect meta-analysis was used when there was no significant heterogeneity.

For CRP, an initial overall meta-analysis was conducted for all included studies. Further sensitive analysis of CRP data was conducted separately for high-quality studies scoring ≥7 on the New-Castle Ottawa scale. In this meta-analysis, we included data for participants with BMI < 30 kg/m^2^ to remove the confounding effect of obesity on CRP. Another sub-group meta-analysis was conducted for studies with CRP data for obese participants (BMI > 30 kg/m^2^). A further sub-analysis was conducted for studies using mg/L as the unit of CRP measurement.

We used the standardized mean difference (SDM) model in this meta-analysis in view of the differences in the units of CRP measurement used across the included studies [151]. In contrast to the mean difference (MD), which is the difference in the means of the study and control groups, the SMD is the MD divided by the standard deviation (SD), derived from either or both of the groups [151]. In addition to being independent of the unit of measurement, the SMD has been found to be more generalizable [152] and an easier way to judge the magnitude of difference between the groups [152]. The general rule described by Cohen et al. suggests that an SMD of 0.2 represents a “small” difference, an SMD of 0.5 represents a “medium” difference, and an SMD of 0.8 represents a “large” difference [153].

## 5. Conclusions

Our meta-analysis provides further strong evidence for a moderate elevation in circulating CRP in PCOS women with a decline in adiponectin, which are indicative of a low-grade chronic inflammation. These changes are mainly attributable to PCOS, independent of, but further accentuated by, obesity. The relevance of this small rise in CRP in relation to the cardiovascular risks remains unknown. Only limited and conflicting data are available on the status IL-6 and TNF-α. Further research is required to investigate the underlying molecular mechanisms of chronic inflammation at the cellular level, especially in adipose tissue.

## Figures and Tables

**Table 1:** Modified Newcastle–Ottawa Scale for risk of bias and quality assessment of the included studies.

Author, Year [ref].	Selection ****	Comparability **	Outcome ***	Overall
Cascella, 2008 [31]	**	**	**	6
Makedos, 2011 [32]	**	**	***	7
Aydogdu, 2012 [33]	***	**	**	7
Tarkun, 2004 [42]	**	**	**	6
Diamanti-Kandarakis, 2006 [43]	***	**	***	8
Topcu, 2006 [44]	**	**	**	6
Moran, 2007 [45]	***	*	**	6
Ruan, 2007 [46]	**	**	**	6
Costa, 2008 [47]	**	**	**	6
Heutling, 2008 [48]	***	*	**	6
Gen, 2008 [49]	**	**	**	6
Jakubowska, 2008 [50]	**	**	**	6
Zwirksa-Korczala, 2008 [51]	***	**	**	7
Çapoğlu, 2009 [52]	***	*	**	6
Kaya, 2009 [53]	***	**	**	7
Chen, 2009 [54]	***	**	***	8
Samy, 2009 [55]	***	**	***	8
Soares, 2009 [56]	***	**	***	8
Verit, 2009 [57]	**	**	**	6
Gowri, 2010 [58]	**	**	**	6
Nikołajuk, 2010 [59]	**	**	**	6
Tan, 2010 [60]	**	**	**	6
TürkçüoĞlu, 2010 [61]	***	*	***	6
Caglar, 2011 [62]	***	*	**	6
Fulghesu, 2011 [63]	**	**	**	6
Hu, 2011 [64]	***	**	***	8
Li, 2011 [65]	***	*	**	6
Lindholm, 2011 [66]	**	*	**	5
Mannerås-Holm, 2011 [67]	***	*	**	6
Ngo, 2011 [68]	**	*	**	5
Torun, 2011 [69]	***	**	***	8
Xiong, 2011 [70]	***	*	**	6
Yang, 2011 [71]	***	*	**	6
Deveer, 2012 [72]	**	**	**	6
Elkholi, 2012 [73]	**	**	*	5
Güdücü, 2012 [74]	***	**	***	8
Iuhas, 2012 [75]	***	**	*	6
Kim, 2012 [76]	**	*	*	4
Rashad, 2012 [77]	****	**	**	8
Tao, 2012 [78]	****	**	**	8
Dehdashtihaghighat, 2013 [79]	***	**	**	7
Xia, 2013 [80]	***	-	*	4
Kahal, 2013 [81]	**	**	**	6
Rey-Roldan, 2013 [82]	**	**	**	6
Tan, 2013 [83]	***	*	**	6
Yildir, 2013 [84]	**	*	***	6
Covington, 2014 [85]	**	*	***	6
Guducu, 2014 [86]	****	**	***	9
Kurt, 2014 [87]	***	**	***	8
Porwal, 2014 [88]	***	**	**	7
Sahin, 2014 [89]	***	**	**	7
Stabile, 2014 [90]	**	*	**	4
Sumithra, 2014 [91]	***	*	**	6
Barcellos, 2015 [92]	**	**	**	6
Daskalopoulos, 2015 [93]	**	*	*	4
Demir, 2015 [94]	***	*	**	6
Güdücü, 2015 [95]	***	**	***	8
Santos, 2015 [96]	***	**	**	7
Sharma, 2015 [97]	***	-	*	4
Yilmaz, 2015 [98]	***	**	***	8
Calan, 2016 [99]	***	**	**	7
Durmus, 2016 [100]	****	**	***	9
Isıka, 2016 [101]	***	*	**	6
Kumar, 2016 [102]	***	**	***	8
Kume, 2016 [103]	***	**	**	7
Mishra, 2016 [104]	****	**	***	9
Ozturk, 2016 [105]	***	**	**	8
Temur, 2016 [106]	***	**	***	8
Victor, 2016 [107]	***	**	***	8
Deng, 2017 [108]	***	**	***	8
Duan, 2017 [109]	***	**	**	7
Abiad, 2018 [110]	**	**	**	6
Kalyan, 2018 [111]	***	**	***	8
Shorakae, 2018 [112]	***	*	**	6
Arpacı, 2019 [113]	***	**	**	7
Demir, 2019 [114]	***	**	***	8
Eken, 2019 [115]	***	**	**	7
Ganie, 2019 [116]	***	*	*	5
Kim, 2019 [117]	***	**	**	7
Maidana, 2019 [118]	***	*	**	6
Sánchez-Ferrer, 2019 [119]	***	*	**	6
Alissa, 2020 [120]	***	*	***	7
Godtfredsen, 2020 [121]	***	*	**	6
González, 2020 [122]	**	*	***	6
Sadeghi, 2020 [123]	***	**	***	8

Asterisks (*) indicate the star rating for each study with a maximum of four stars for selection, two for comparability, and three for outcome criteria.

**Table 2:** Characteristics and CRP results of 85 studies included in the systematic review and meta-analysis.

Author, Year [ref]	Country	Study Design	PCOS (*n*)	Control (*n*)	Hs CRP Kit	Strata	CRP PCOS	CRP Control	*p*	CRP Change	CRP Unit	Age & BMI Matching or Adjusting
Cascella, 2008 [31]	Turkey	Case–control	200	100	Not specified	Nil	1.9 ± 0.8	0.8 ± 0.4	<0.001	↑	mg/L	Both matched
Makedos, 2011 [32]	Greece	Case–control	188	43	INA	Nil	0.55 ± 0.08	0.27 ± 0.08	<0.001	↑	mg/L	Both matched
Aydogdu, 2012 [33]	Turkey	Case–control	40	40	Hs-EIA	Nil	1.9 ± 1.6	1.0 ± 1.0	0.002	↑	mg/L	Both matched
Tarkun, 2004 [42]	Turkey	Case–control	37	25	CLIA	Nil	<0.25>	<0.09>	<0.007	↑	mg/dl	Both matched
Diamanti-Kandarakis, 2006 [43]	Greece	Interventional	62	45	Hs-ELISA	Nil	1.31 ± 0.22	0.92 ± 0.27	0.005	↑	mg/dl	Both matched
Topcu, 2006 [44]	Turkey	Case–control	28	26	ITA	Nil	2.5 ± 1.8	1.7 ± 1.2	>0.05	NSD	mg/dl	Both matched
Moran, 2007 [45]	Australia	Case–control	17	15	ITA	BMI > 35	5.5 ± 3.1	4.9 ± 3.0	0.603	NSD	mg/dl	BMI matched Age, not matched
Ruan, 2007 [46]	China	Case–control	74	51	ITA	Nil	((2.13))	((0.86))	<0.001	↑	mg/L	Both matched
Costa, 2008 [47]	Brazil	Case–control	57	37	ITA	Nil	3.1 ± 3.2	1.4 ± 0.86	0.004	↑	mg/L	Both matched
Heutling, 2008 [48]	Turkey	Interventional	21	15	ITA	Nil	4.43 ± 2.02	3.74 ± 1.69	>0.05	NSD	mg/dl	Both matched
Gen, 2008 [49]	Germany	Case–control	83	39	ITA	Nil	3.26 ± 2.33	1.61 ± 1.36	<0.001	↑	mg/L	Both matched
Jakubowska, 2008 [50]	Poland	Interventional	29	29	INA	Nil	3.53 ± 3.64	3.86 ± 4.27	>0.05	NSD	mg/L	Both matched
Zwirksa-Korczala, 2008 [51]	Poland	Case–control	40	20	ITA	Lean Obese	1.8 ± 0.3 3.4 ± 1.6	0.5 ± 0.1	<0.01 <0.02	↑	mg/L	Age matched; BMI matched (lean)
Çapoğlu, 2009 [52]	Turkey	Case–control	46	20	ITA	Nil	2.0 ± 4.2	0.9 ± 1.0		NSD **	mg/L	Both matched
Kaya, 2009 [53]	Turkey	Case–control	60	60	ITA	Nil	4.4 ± 1.6	1.5 ± 0.6	<0.01	↑	mg/dl	Both matched
Chen, 2009 [54]	Taiwan	Case–control	155	37	ITA	Nil	2.31 ± 0.51 (SE)	0.97 ± 0.2 (SE)	0.0159	↑	mg/L	Both matched
Samy, 2009 [55]	Egypt	Case–control	108	75	Hs-ELISA	BMI < 27 BMI ≥ 27	1.67 ± 0.23 3.45 ± 0.35	1.04 ± 0.25 1.15 ± 0.22	<0.0001	↑	mg/L	Both matched
Soares, 2009 [56]	Brazil	Case–control	40	50	CLIA	Nil	2.03 ± 2.7	3.32 ± 3.72	0.97	NSD	mg/L	Both matched
Verit, 2009 [57]	Turkey	Case–control	52	48	Spectro-photometry	Nil	1.6 ± 0.6	0.4 ± 0.5	<0.0001	↑	mg/L	Both matched
Gowri, 2010 [58]	Oman	Case–control	57	57	ITA	Nil	5.41 ± 4.84	4.76 ± 4.54	0.461	NSD	mg/L	Both matched
Nikołajuk, 2010 [59]	Poland	Case–control	78	34	ITA	Lean Obese	0.67 ± 0.50 1.16 ± 0.74	0.47 ± 0.22 1.20 ± 0.83	>0.5	NSD	mg/L	Both matched
Tan, 2010 [60]	UK	Case–control	21	39	ITA	Nil	3.3 (2.4–5.5)	1.3 (0.5–2.5)	<0.01	↑	mg/L	Both matched
TürkçüoĞlu, 2010 [61]	Turkey	Case–control	91	51	INA	Nil	5.1 +3.9	3.5 ± 1.3	0.004	↑	mg/L	Both matched
Caglar, 2011 [62]	Turkey	Case–control	61	21	Not specified	Nil	0.6 [0.2–4.6]	1.3 [0.2–18.6]	<.05	↑	mg/L	Both matched
Fulghesu, 2011 [63]	Italy	Case–control	44	55	Hs-ELISA	IR PCOS	0.55 [0.05–1.66]	0.139 [0.04–1.77]	<0.005	↑ ***	ng/ml	Both matched
Hu, 2011 [64]	China	Case–control	85	65	INA	Nil	1.9 ± 2.0	0.9 ± 0.8	0.009	↑	mg/L	Both matched
Li, 2011 [65]	UK	Case–control	27	25	ITA	Nil	2.2 [0.2–14.5]	0.8 [0.1–9.6]	0.017	NSD *	mg/L	Age, BMI adjusted
Lindholm, 2011 [66]	Sweden	Case–control	30	20	ITA	Lean OWT	1.10 ± 0.99 4.31 ± 3.16	- 3.48 ± 4.06	- >0.5	NSD	mg/L	Age, matched BMI, matched (obese)
Mannerås-Holm, 2011 [67]	China	Case–control	70	30	ITA	Nil	1.96 ± 1.92	1.00 ± 1.11	0.598	NSD *	mg/L	Both matched
Ngo, 2011 [68]	Australia	Case–control	27	20	Not specified	Nil	1.5 <0.25, 5.7>	0.4 <0.1–18>	0.008	↑	mg/L	Both matched
Torun, 2011 [69]	Turkey	Case–control	30	20	Not specified	Nil	0.22 ± 0.2	0.13 +0.1	0.3	NSD	mg/L	Both matched
Xiong, 2011 [70]	Chin	Case–control	86	51	CLIA	Nil	2.53 ± 2.97	0.95 ± 1.14	0.001	↑	mg/L	Both matched
Yang, 2011 [71]	China	Case–control	133	116	ITA	Nil	0.49 ± 0.88	1.46 ± 2.29	<0.05	↑	mg/L	Both matched
Deveer, 2012 [72]	Turkey	Case–control	25	25	ITA	Nil	1.0 [1.0–12.0]	1.0 [1.0–19.0]	0.286	NSD	mg/L	Both matched
Elkholi, 2012 [73]	Egypt	Case–control	63	45	INA	NIR GO IR AO	0.19 ± 0.01 0.55 ± 0.10	0.18 ± 0.04 0.52 ± 0.07	>0.05	NSD	mg/dl	Both matched
Güdücü, 2012 [74]	Turkey	Case–control	62	40	ITA	Nil	0.27 ± 0.28	0.19 ± 0.25	0.046	↑	mg/L	Both matched
Iuhas, 2012 [75]	Romania	Case–control	31	29	Not specified	Nil	3.22 ± 0.76	3.33 ± 0.83	0.60 *	NSD *	mg/L	Both matched
Kim, 2012 [76]	Korea	Case–control	115	103	Not specified	Nil	0.24 ± 0.67	0.14 ± 0.46	0.231	NSD **	mg/L	Age, not matched BMI, matched
Rashad, 2012 [77]	Egypt	Case–control	107	92	Hs-ELISA	Nil	1.0 ± 0.19	0.79 ± 0.1	<0.05	↑	mg/L	Both matched
Tao, 2012 [78]	China	Case–control	137	123	INA	Lean Obese	2.80 {2.24–3.36} 4.56 {3.94–5.18}	1.08 {0.93–1.23} 3.37 {2.69–4.04}	0.0001	↑	mg/L	Both matched
Dehdashtihaghighat, 2013 [79]	Iran	Case–control	42	42	ITA	Nil	4.7 ± 1.5	0.00	0.039	↑	mg/L	Both matched
Xia, 2013 [80]	China	Case–control	59	57	ITA	Nil	1.58 ± 0.63	1.07 ± 0.47	<0.01	↑	mg/L	Both matched
Kahal, 2013 [81]	UK	Case–control	19	17	Not specified	Nil	6.2 ± 8.7	5.1 ± 3.4	>0.05	NSD	mg/L	Both matched
Rey-Roldan, 2013 [82]	Argentina	Case–control	20	20	ITA	Nil	<<5.1>>	<<0.8>>	<0.0001	↑	mg/L	Both matched
Tan, 2013 [83]	UK	Case–control	83	39	ITA	Nil	2.8 (1.5–4.6)	1.3 (0.6–2.3)	<0.01	↑	mg/L	Both matched
Yildir, 2013 [84]	Turkey	Case–control	34	20	INA	Nil	4.75 ± 3.99	3.76 ± 1.83	0.151	NSD	mg/L	Both matched
Covington, 2014 [85]	USA	Interventional	8	8	CLIA	Nil	3.8 ± 5.2	4.6 ± 6.1	0.79	NSD	mg/L	Both matched
Guducu, 2014 [86]	Turkey	Case–control	58	34	ITA	Nil	2.21 ± 3.8	1.69 ± 3.56	0.174	NSD	mg/L	Both matched
Kurt, 2014 [87]	Turkey	Case–control	62	60	INA	Obese Lean	6.0 ± 0.8 2.8 ± 0.6	3.4 ± 0.6 2.8 ± 0.6	<0.001 <0.001	↑	mg/L	Both matched
Porwal, 2014 [88]	India	Case–control	41	40	ITA	Nil	3.76 ± 3.60	1.38 ± 1.48	<0.05	↑	mg/L	Both matched
Sahin, 2014 [89]	Turkey	Case–control	91	53	ITA	Nil	0.43± 0.67	0.15± 0.25	0.007	↑	mg/L	Both matched
Stabile, 2014 [90]	Italy	Interventional	15	15	ITA	Nil	0.27± 0.19	0.11 ± 0.1	<0.01	↑	mg/L	Both matched
Sumithra, 2014 [91]	Greece	Case–control	61	61	ITA	Nil	4.35 ± 4.16	0.40 ± 0.22	<0.001	↑	mg/L	Age Matched BMI adjusted
Barcellos, 2015 [92]	Brazil	Case–control	25	23	Hs-ELISA	Nil	0.9 [0.1–5.7]	0.5 [0.1–4.6]	0.361	NSD	mg/L	Both matched
Daskalopoulos, 2015 [93]	Greece	Case–control	240	60	CLIA	PHEN 1 PHEN 2 PHEN 3 PHEN 4	9.01 ± 0.85 4.13 ± 0.68 3.5 ± 0.82 2.98 ± 0.6	3.09 ± 0.53	>0.05	NSD	mg/L	Both matched
Demir, 2015 [94]	Turkey	Case–control	35	35	ITA	Nil	0.3 ± 0.4	0.1 ± 0.2	0.001	↑	mg/L	Both matched
Güdücü, 2015 [95]	Turkey	Case–control	35	30	ITA	Nil	2.03 ± 3.59	1.68 ± 3.76	0.197	NSD	mg/L	Both matched
Santos, 2015 [96]	Brazil	Case–control	20	20	CLIA	BMI < 25 BMI > 25	2.63 ± 0.65 2.35 ± 0.55	0.63 ± 0.37 0.82 ± 0.48	0.01 0.01	↑	mg/L	Both matched
Sharma, 2015 [97]	India	Case–control	100	50	Hs-ELISA	Nil	2.17 ± 0.30	2.27 ± 0.95	0.74	NSD	mg/L	Both adjusted
Yilmaz, 2015 [98]	Turkey	Case–control	41	30	INA	Overall Obese Lean	2.78 ± 3.20 4.06 ± 3.52 0.77 ± 2.66	2.58 ± 3.09 4.037 ± 3.18 1.31 ± 2.44	0.545 0.965 0.926	NSD	mg/L	Both matched
Calan, 2016 [99]	Turkey	Case–control	90	80	ITA	Nil	1.49 ± 0.50	0.56 ± 0.49	<0.001	↑	m/L	Both matched
Durmus, 2016 [100]	Turkey	Case–control	76	38	INA	Overall BMI < 25 BMI > 25	0.9 [0.16–14.9] 0.57 [0.16–14.9] 2.24 [0.33–14.3]	0.74 [0.16–10.0] 0.36 [0.16–2.60] 1.43 [0.16–10.0]	0.081 0.118 0.137	NSD	mg/dl	Both matched
Isıka, 2016 [101]	Turkey	Case–control	41	32	ITA	Nil	5.46 ± 4.72	2.86 ± 4.59	<0.001	↑	mg/L	Both matched
Kumar, 2016 [102]	India	Case–control	80	80	ITA	Nil	8.5 ± 2.7	1.9 ± 1.2	0.0001	↑	mg/L	Both matched
Kume, 2016 [103]	Turkey	Case–control	152	152	ITA	Nil	0.76 ± 0.34	0.47 ± 0.22	<0.001	↑	mg/L	Both matched
Mishra, 2016 [104]	India	Case–control	44	45	ITA	Nil	4.21 ± 3.5	2.72 ± 1.78	<0.05	↑	mg/L	Both matched
Ozturk, 2016 [105]	Turkey	Case–control	53	40	ITA	Nil	2.1 ± 1.7	1.7 ± 1.0	0.095	NSD	mg/L	Both matched
Temur, 2016 [103]	Turkey	Case–control	38	41	Not specified	Nil	3.15 ± 2.23	1.18 ± 1.47	<0.001	↑	mg/L	Both matched
Victor, 2016 [107]	Spain	Case–control	101	105	INA	IR NIR	3.29± 3.42 3.72 ± 3.19	2.07 ± 2.46	0.018 0.137 *	NSD	mg/L	Both matched
Deng, 2017 [108]	China	Case–control	30	0	ITA	Nil	1.05 (0.218, 2.720)	0.19 (0.055–0.618)	<0.001	↑	mg/L	Both matched
Duan, 2017 [109]	China	Interventional	90	85	ITA	Nil	1.99 ± 2.12	0.89 ± 0.65	0.047	↑	mg/L	Age, matched BMI adjusted
Abiad, 2018 [110]	Lebanon	Case–control	6	16	Spectro-photometry	BMI > 40	10.04 ± 5.85	6.63 ± 4.12	0.203	NSD	mg/L	Both matched
Kalyan, 2018 [111]	Bahrain	Case–control	200	119	INA	Nil	11.1 ± 1.1	15.5 ± 1.6	0.000	↑	mg/L	Both matched
Shorakae, 2018 [112]	Australia	Case–control	49	22	ITA	Nil	2.2 (3)	4.4 (6)	>0.05	↑	mg/L	Both matched
Arpacı, 2019 [113]	Turkey	Case–control	38	48	ITA	Nil	0.49 ± 0.55	0.18 ± 0.19	0.025	↑	mg/L	Both matched
Demir, 2019 [114]	Turkey	Case–control	80	80	ITA	Nil	1.22 ± 0.57	0.67 ± 0.20	<0.001	↑	mg/L	Both matched
Eken, 2019 [115]	Turkey	Case–control	69	70	Not specified	Overall NWT Obese	1.08 ± 0.83 0.79 ± 0.39 1.38 ± 1.04	0.46 ± 0.46 0.39 ± 0.36 0.55 ± 0.55	0.001	↑	mg/dl	Both matched
Ganie, 2019 [116]	India	Case–control	82	179	Hs-ELISA	Veg Non-veg	3.83 ± 1.68 2.38 ± 0.88	2.19 ±1.48 1.68 ± 1.52	<0.01 <0.01	↑	mg/L	Both matched
Kim, 2019 [117]	Korea	Case–control	26	59	LIA	Nil	0.03 (0.02–0.08)	0.04 (0.02–0.09)	0.420	NSD	mg/dl	Both matched
Maidana, 2019 [118]	Argentina	Case–control	73	33	ITA	Nil	2.38 (0.36–14.53)	11 (0.27–9.30)	0.005	NSD *	mg/L	Both adjusted
Sánchez-Ferrer, 2019 [119]	Spain	Case–control	126	159	ITA	Nil	0.30 (0.16–0.44)	0.24 (0.20–0.30)	0.03	NSD *	mg/dl	Both adjusted
Alissa, 2020 [120]	KSA	Case–control	82	82	ITA	Nil	2.10 ± 0.4	5.1 ± 0.7	<0.0001	↑	mg/L	Both matched
Godtfredsen, 2020 [121]	Denmark	Case–control	90	35	INA	Nil	1.0 (0.43–2.2)	0. 59 (0.29–1.23)	0.01	↑	mg/L	Both matched
González, 2020 [122]	USA	Interventional	14	14	Hs-ELISA	Lean Obese	1.8 ± 0.2 8.9 ± 2.0	0.3 ± 0.1 6.6 ± 1.5	<0.05 <0.02	↑	mg/L	Both matched
Sadeghi, 2020 [123]	Iran	Case–control	120	60	Hs-ELISA	Nil	4.00 ± 1.23	2.46 ± 0.95	<0.01	↑	mg/ml	Both matched

CRP data presented as mean ± sd, <geometric mean>, median (IQR), median [range], median <unspecified range>, ((median)); mean {95% confidence interval} or <<average>>. Study design: for interventional studies, baseline pre-treatment CRP data are presented in this table. **↑** significantly elevated in PCOS women. * after adjustment for BMI. ** Trend towards increase, which is not statistically significant. *** elevated only in PCOS women with insulin resistance. **Abbreviations**: **Hs**, high sensitivity; **ELISA**, enzyme-linked immunosorbent assay; **CLIA**, chemiluminescence immunoassay; **ITA**; immunoturbidimetric assay; **INA**, immunonephelometric assay; **LIA**, latex immunoassay; **IR**, insulin resistant; **NIR**; non-insulin resistant; **NSD**, no statistically significant difference; **NIR GO**, non-insulin resistant and gynaecoid obesity, **IR AO**: insulin resistant and android obesity; **Veg**, vegetarian; **OWT**, overweight; **NWT**, normal weight; **PHEN**, PCOS phenotype.

**Table 3:** Characteristics of 45 studies with other inflammatory markers.

Author, Year	Country	PCOS NO	Controls NO	CRP	IL-6	TNF	Adiponectin	Other Markers		Age, BMI Matching
								Marker	level	
Diamanti-Kandarakis, 2006 [30]	Greece	62	45	↑	–	–	–	sVCAM sICAM, sE-Selectin	NSD ↑ ↑	Age, BMI matched
Cascella, 2008 [31]	Turkey	200	100	↑	–	–	–	PAI-1 WCC	↑ ↑	Age, BMI matched
Aydogdu, 2012 [33]	Turkey	40	40	↑	–	–	–	PTX-3	↑	Age, BMI matched
Moran, 2007 [45]	Australia	17	15	NSD	NSD	NSD	NSD	–	–	PCOS older, BMI > 35
Ruan, 2007 [46]	China	74	51	↑	–	–	–	WCC	↑	Age, BMI matched
Heutling, 2008 [48]	Germany	83	39	↑	NSD	–	–	ADMA SDMA	↑ NSD	Age, BMI matched
Gen, 2008 [49]	Turkey	21	15	NSD	NSD	–	–	Visfatin	↑	Age, BMI matched
Jakubowska, 2008 [50]	Poland	29	29	NSD	NSD	↑	↓	-		Age, BMI matched
Zwirksa-Korczala, 2008 [51]	Poland	40	20	↑	–	–	↓	CD40L Visfatin, Resistin, sP-Selectin sE-Selectin	↑ ↑ ↑ ↑ ↑	Age, BMI matched
Çapoğlu, 2009 [52]	Turkey	46	20	NSD	–	–	–	Resistin	↓	Age, BMI matched
Kaya, 2009 [53]	Turkey	60	60	↑	↑	–	–	Il-18	↑	Age, BMI matched
Samy, 2009 [55]	Egypt	108	75	↑	↑	↑	–	–	–	Age matched; BMI stratified
Soares, 2009 [56]	Brazil	40	50	NSD	NSD	NSD	–	–	–	Age, BMI matched
Nikołajuk, 2010 [59]	Poland	78	34	NSD	NSD	–	–	SPG130 sIL-6R	↑ ↓	Age, BMI matched
Tan, 2010 [60]	UK	21	39	↑	–	–	–	Omentin	↑	Age, BMI matched
Fulghesu, 2011 [63]	Italy	44	55	↑	NSD	↑	↓	Leptin TGF IL-B	↑ NSD NSD	Age, BMI matched
Hu, 2011 [64]	China	85	65	↑	–	–	–	MCP-1	↑	
Mannerås-Holm, 2011 [67]	China	70	30	NSD*	NSD	NSD	–	PAI-1	↑	Age, BMI matched
Ngo, 2011 [68]	Australia	27	20	↑	–	–	–	Vit. D	↓	Age, BMI matched
Torun, 2011 [69]	Turkey	30	20	NSD	–	–	–	TAS TOS OSI LOOH	NSD ↑ ↑ ↑	Age, BMI Matched
Xiong, 2011 [70]	China	86	51	↑	↑	↑	–	WCC Neutrophil count Lymphocyte count Monocyte count Eosinophil count	NSD NSD ↑ ↑ ↑	Age, BMI matched
Yang, 2011 [71]	China	133	116	↑	–	–	–	C3	↑	Age, BMI adjusted
Elkholi, 2012 [73]	Egypt	63	45	↑	↑	–	↓	IL-10 PAI-1	↓ ↓	Age, BMI matched
Rashad, 2012 [77]	Egypt	107	92	↑	–	–	–	WCC Neutrophil count	↑ ↑	Age, BMI matched
Dehdashtihaghighat, 2013 [79]	Iran	42	42	↑	–	–	–	C3	NSD	Age, BMI matched
Xia, 2013 [80]	China	59	57	↑	–	–	–	IL-1β IL-1Ra	↑ ↑	Age, BMI matched
Rey-Roldan, 2013 [82]	Argentina	20	20	↑	NSD	–	–	IL-6 D	NSD	Age, BMI matched
Tan, 2013 [83]	UK	83	39	↑	–	NSD	NSD	Leptin Visfatin	NSD NSD	Age, BMI matched
Covington, 2014 [85]	USA	8	8	NSD	–	–	–	WCC	↑	Age, BMI matched
Guducu, 2014 [86]	Turkey	58	34	NSD	–	–	–	PTX-3	↓	Age, BMI matched
Kurt, 2014 [87]	Turkey	62	60	↑	–	–	–	WCC Neutrophil count Lymphocyte count NLR	↑ ↑ NSD ↑	Age matched; BMI stratified
Barcellos, 2015 [92]	Brazil	25	23	NSD	NSD	NSD	–			Age matched; BMI stratified
Güdücü, 2015 [95]	Turkey	35	30	NSD	–	–	–	Chemerin	NSD	Age matched, BMI adjusted
Santos, 2015 [96]	Brazil	20	20	↑	NSD	NSD	–	–		Age matched; BMI stratified
Yilmaz, 2016 [98]	Turkey	41	30	↑ lean	–	–	–	WCC Neutrophil count Lymphocyte count Monocyte count	NSD ↑ NSD NSD	Age, BMI adjusted
Calan, 2016 [99]	Turkey	90	80	↑	–	–	–	MIF	↑	Age, BMI matched
Isıka, 2016 [101]	Turkey	41	32	↑	–	–	–	WCC XO SOD NLR	↑ ↑ ↑ NSD	Age, BMI matched
Kume, 2016 [103]	Turkey	152	152	↑	–	↑	–	–		Age, BMI matched
Ozturk, 2016 [105]	Turkey	53	40	↑	–	–	–	GGT IMA	↑ NSD	Age, BMI matched
Deng, 2017 [108]	China	30	0	↑	–	–	–	IP-10	↑	Age, BMI matched
Shorakae, 2018 [112]	Australia	49	22	↑	–	–	↓	–		Age, BMI matched
Arpacı, 2019 [113]	Turkey			↑	–	–	–	Neuregulin-1	↑	Age, BMI matched
Demir, 2019 [114]	Turkey	80	80	↑	–	–	–	Fractalkine	↑	Age, BMI matched
Ganie, 2019 [116]	India	82	179	↑	↑	↑	↓	IL-β IL-10 Resistin	↑ ↓ ↑	Age, BMI matched
Sadeghi, 2020 [123]	Iran	120	60	↑	–	–	↓	CTRP6 b	↑	Age, BMI matched

**↑** significantly elevated in PCOS women, * after adjustment for BMI. **↓** significantly reduced in PCOS women. Abbreviations: **NSD**, no significant difference between PCOS and control; **sVCAM**, soluble vascular cell adhesion molecule; **sICAM**, soluble intercellular adhesion molecule; **ADMA**, Asymmetrical dimethylarginine; **SDMA**, **WCC**, white cell count; Symmetrical dimethylarginine; **sE-selectin**, soluble endothelial leukocyte adhesion molecule; **PAI-1**, plasminogen-activated inhibitor; **SPG**, soluble glycoprotein; **sIL-6R**, soluble IL-6 receptor; **TGF**, transforming growth factor; **Vit**, Vitamin; **MCP-1**, monocyte chemoattractant protein 1; **TAS**, total antioxidant status; **TOS**, total oxidative stress, **OSI**, oxidative stress index, **LOOH**, lipid hydroperoxide; **C3**, Complement C3; **PTX-3**, Pentraxin 3; **MIF**, Macrophage migration inhibitory factor; **XO**, xanthine oxidase activity, **SOD**, superoxide dismutase activity; **NLR**, neutrophil lymphocyte ratio; **GGT**, gamma-glutamyl transferase; **IMA**, Ischaemia-modified albumin; **OS**, oxidative stress; **IP-10**, Interferon g-induced protein 10 kDa; **CTRP6**, C1q/TNF-α-related protein 6.

## Data Availability

Not applicable.
